# miR-1248 enhances bortezomib-induced autophagy by targeting MEF2C/p38-MAPK signaling in multiple myeloma

**DOI:** 10.3389/fonc.2026.1847922

**Published:** 2026-06-26

**Authors:** Wei Wang, Rong-juan Zhang, Ming-shuai Ma, Xiao-min Shi, Qing Zhang, Chong Li, Zhi-hua Zhang, Chang-lai Hao

**Affiliations:** Department of Hematology, Affiliated Hospital of Chengde Medical College, Chengde, Hebei, China

**Keywords:** apoptosis, autophagy, bortezomib, MAPK pathway, MEF2C, miR-1248, multiple myeloma

## Abstract

**Objective:**

Multiple myeloma (MM) is incurable and many patients respond poorly to bortezomib. New strategies to improve bortezomib sensitivity are needed.

**Methods:**

We screened bortezomib-responsive miRNAs by RNA-seq in RPMI8226 cells (fold change >4, P<0.05). miR-1248 was measured by qRT-PCR in MM cells and in plasma from 30 patients before/after VCD therapy. MEF2C was examined by qRT-PCR and Western blot. A dual-luciferase reporter assay was performed to test miR-1248 binding to the MEF2C 3′-UTR. After miR-1248 inhibitor transfection, we detected MEF2C, p38 and autophagy markers by qRT-PCR/Western blot and assessed autophagic activity by staining. Rescue experiments overexpressed MEF2C. *In vivo*, a xenograft model (n=6) was used to detect these markers after miR-1248 inhibition plus bortezomib(0.5 mg/kg).

**Results:**

Bortezomib increased miR-1248 by 2-fold (P<0.05) and reduced MEF2C mRNA by 62% and protein by 55% (both P<0.01). Plasma miR-1248 also rose after VCD therapy (median 2.1-fold, P<0.05). Luciferase assays confirmed direct targeting of the MEF2C 3′-UTR by miR-1248. Inhibiting miR-1248 reduced bortezomib-induced autophagy markers and staining, and partially restored MEF2C and p38 (P<0.05). MEF2C overexpression weakened bortezomib-induced apoptosis (cell viability rose from 42 ± 5% to 71 ± 6%, P<0.01) and suppressed those markers. In xenografts, miR-1248 knockdown cut tumor inhibition from 58 ± 7% to 23 ± 9% (P<0.01), with partial restoration of MEF2C/p38 and blunted autophagy marker elevation.

**Conclusions:**

Our findings indicate that miR-1248 enhances bortezomib sensitivity in MM cells by directly targeting MEF2C, accompanied by reduced p38 expression and autophagic activity. The coordinated changes in MEF2C and p38 define a functional axis that may be targeted to improve bortezomib response.

## Introduction

Multiple myeloma (MM) remains incurable. The proteasome inhibitor bortezomib has improved patient outcomes, but many patients still respond poorly (1–10). Improving bortezomib sensitivity is therefore a key clinical goal.

Autophagy, a conserved lysosomal degradation pathway, helps maintain cellular homeostasis by removing misfolded proteins and damaged organelles (11). In MM cells, which often accumulate toxic immunoglobulin aggregates, autophagy can serve as a survival mechanism (12). Bortezomib has been shown to modulate autophagy, with outcomes ranging from cytoprotection to cell death depending on context. In this study, we focus on bortezomib-induced autophagy that may enhance drug sensitivity rather than promote resistance.

MicroRNAs regulate gene expression post-transcriptionally (13). In MM, miRNA expression profiles are frequently altered and linked to tumor progression, chemoresistance and autophagy (14, 15). To identify miRNAs involved in bortezomib response, we performed RNA-seq on bortezomib-treated RPMI8226 cells. Among the differentially expressed miRNAs, miR-1248 showed the most significant upregulation (fold change >4, P<0.05). This miRNA is located at 3q27.3. It has been reported to play opposing roles in different cancers: it promotes lung cancer but suppresses gastric cancer (16–18). However, its expression and function in MM, especially in bortezomib sensitivity and autophagy, remain unknown.

Transcriptome analysis pointed to MEF2C as a potential target of miR-1248. We also found that p38 MAPK expression changed along with MEF2C after modulating miR-1248. Both MEF2C and p38 are known to play roles in stress responses and cell survival in other systems (19–22), but whether they are involved in autophagy regulation is not well established, especially in MM. We therefore asked if miR-1248 affects bortezomib sensitivity by coordinately regulating MEF2C and p38, and whether this involves changes in autophagic activity.

In this study, we identify miR-1248 as a previously unrecognized regulator of bortezomib sensitivity in MM. We demonstrate that miR-1248 directly targets MEF2C, and that this is accompanied by reduced p38 expression and suppression of autophagic activity. These findings define a coordinated miR-1248–MEF2C–p38MAPK axis that may serve as a potential therapeutic target to improve bortezomib response in MM.

## Materials and methods

### Genomic screening and target prediction

RPMI8226 cells (three biological replicates) were treated with 6 nM bortezomib for 24 h. RNA integrity was confirmed (RIN >7.0). Stranded RNA-seq libraries were prepared and sequenced on the DNBSEQ platform (paired-end, ~30 M reads/sample). Reads were aligned to hg38 with STAR v2.7, and gene counts obtained with featureCounts. Differential expression was analyzed with DESeq2 (|FC|>4 or <0.25, P<0.05, FDR<0.05, Benjamini-Hochberg). Putative miR-1248 targets were predicted by miRDB and TargetScan, then cross-referenced with RNA-seq data. MEF2C was selected because it was predicted by both databases, its mRNA decreased after bortezomib (while miR-1248 increased), and it is involved in stress signaling. Candidate expression was validated by qRT-PCR.

### Patients and sample collection

Peripheral blood was collected from 30 newly diagnosed MM patients before and after one cycle of VCD chemotherapy (bortezomib, cyclophosphamide, dexamethasone). All participants gave informed consent; ethics approval No. CYFYLL2024049. Plasma was separated by centrifugation (300 × g, 15 min, 20 °C) and stored at −80 °C. RNA was extracted using a miRNA isolation kit (TIANGEN). Paired pre-/post-treatment samples were analyzed.

### Cell culture, transfection and infection

RPMI8226, U266 and 293T cells (ATCC) were tested mycoplasma-negative (PCR) and used within 15 passages. RPMI8226 and U266 were cultured in RPMI-1640, 293T in DMEM, both with 10% FBS. For miR-1248 inhibition, cells were transfected with 50 nM inhibitor or NC (GenePharma) using Lipofectamine 3000, then treated with 6 nM bortezomib for 24 h. For luciferase assay, 293T cells were co-transfected with miR-1248 mimic or NC (50 nM) and wild-type/mutant MEF2C 3′-UTR plasmids (Gikegene) using Lipofectamine 3000. For rescue, cells were infected with MEF2C-overexpressing lentivirus (GenePharma) at MOI 90–110. All transfections were in triplicate. 

### RNA extraction and qRT- PCR

Total RNA was extracted with TRIzol (Invitrogen). Reverse transcription used Tiangen kits. qRT-PCR was run on an ABI 7500. miR-1248 was normalized to U6; MEF2C, p38, Beclin1, LAMP1, LC3B to GAPDH. Relative expression was calculated by 2^-^ΔΔCt. Primers are listed in **Table 1**. Three biological replicates, each with three technical repeats.

**Table 1:** Sequences of primers used in qRT-PCR.

Factor	Primer sequence	Amplified fragment size(bp)
LC3	F GCCTTCTTCCTGCTGGTGAACC R TCCTCGTCTTTCTCCTGCTCGTAG	86
LAMP1	F CCACAGTCGGCAATTCCTACAAG R CCAGCAGACACTCCTCCACAG	144
GAPDH	F GCACCGTCAAGGCTGAGAAC R TGGTGAAGACGCCAGTGGA	138
Beclin1	F CGCCGTCTTGAACCAGCTATCC R ACTTGTCCCAGATTCTGCACCTTTG	121
MEF2C	F GAGCGTGCtGTGTGACTGTGAG R CATGTCGGTGCTGGCaTACTGG	82
P38	F TGGTGTGCTCTGCTTATGATAATGTC R AGTAGGTCTGGTGCTCAAAGGG	81

### Luciferase reporter assay

After 48 h co-transfection, cells were lysed with reporter lysis buffer (Promega E397A). Firefly luciferase activity was measured and normalized to Renilla using the dual-luciferase system (Promega E1910).

### Western blot

Cells or tumor tissues were lysed in RIPA with phosphatase inhibitors (Solarbio R0020). Protein (20 µg/lane) was separated on 12% SDS-PAGE, transferred to PVDF membranes (Millipore IPVH00010), and blocked with 5% skim milk. Primary antibodies: anti-LC3 (Abcam ab192890, 1:1000), anti-LAMP1 (ab25630, 1:1000), anti-MEF2C (ab78888, 1:1000), anti-p38 total (ab170099, 1:1000), anti-Beclin1 (ab207612, 1:1000), and anti-GAPDH (1:5000). Secondary: HRP-conjugated (Abcam ab6721, 1:5000). Bands were visualized by ECL (Gene Flow) and quantified with ImageJ. LC3-I and LC3-II were separately quantified. Three independent experiments.

### Assessment of autophagic activity

Acridine orange (Beyotime C0233) and Lyso-Tracker Red (MesGen MF8123) staining were performed per the manufacturers’ protocols. Stained cells were examined by fluorescence microscopy (excitation 488/515 nm for AO, 577/590 nm for Lyso-Tracker). These assays detect acidic vesicles and lysosomes, not autophagic flux.

### Cell viability

CCK-8 assay (Dojindo CK04) was used. After rescue, cells were seeded in 96-well plates (7×10^3^ cells/well, five replicates). After 24 h bortezomib treatment, 10 µL CCK-8 was added for 2 h at 37 °C. Absorbance at 450 nm was read. Viability was calculated relative to OE-NC untreated (100%). Three independent experiments.

### Xenograft model

Male BALB/c nude mice (5-6 weeks old, n=6/group) were injected subcutaneously with 5×10^6^ RPMI8226 cells expressing miR-1248 inhibitor or NC (200 µL PBS, no Matrigel). When tumors became palpable (~2 weeks), mice were randomly assigned to four groups: NaCl+NC, Bor+NC, NaCl+miR-1248 inhibitor, Bor+miR-1248 inhibitor. Bortezomib (0.5 mg/kg) or saline was injected intraperitoneally on days 1,4,8,11. Tumor volume was measured weekly with calipers and calculated as (length×width²)/2. Body weight was monitored twice weekly. Humane endpoint: tumor >2000 mm³, ulceration, or weight loss >20%. Mice were euthanized by CO_2_ inhalation followed by cervical dislocation. The investigator measuring tumors was blinded to group. At day 35, tumors were excised and snap-frozen in liquid nitrogen.

### Statistical analysis

Data are mean ± SD from ≥3 biological replicates. Normality (Shapiro-Wilk) and equal variance (Levene) were checked. Two-group comparisons used two-tailed t-test (unpaired for cells, paired for patient plasma). Multiple groups were compared by one-way ANOVA with Tukey’s *post hoc* test. Repeated tumor measurements were analyzed by mixed-effects model (Geisser-Greenhouse). P<0.05 was significant. GraphPad Prism 9.0 was used.

## Result

### RNA sequencing identifies miR-1248 as a bortezomib-responsive miRNA targeting the MEF2C/MAPK axis in MM

To identify miRNAs responsive to bortezomib, we performed RNA sequencing on RPMI8226 cells before and after treatment. Differentially expressed miRNAs were identified using thresholds of |fold change| >4 and adjusted P < 0.05. miR-1248 showed the most significant upregulation (fold change = 4.3, adjusted P = 0.008) and was selected based on fold change and statistical significance.

**Figure 1:**
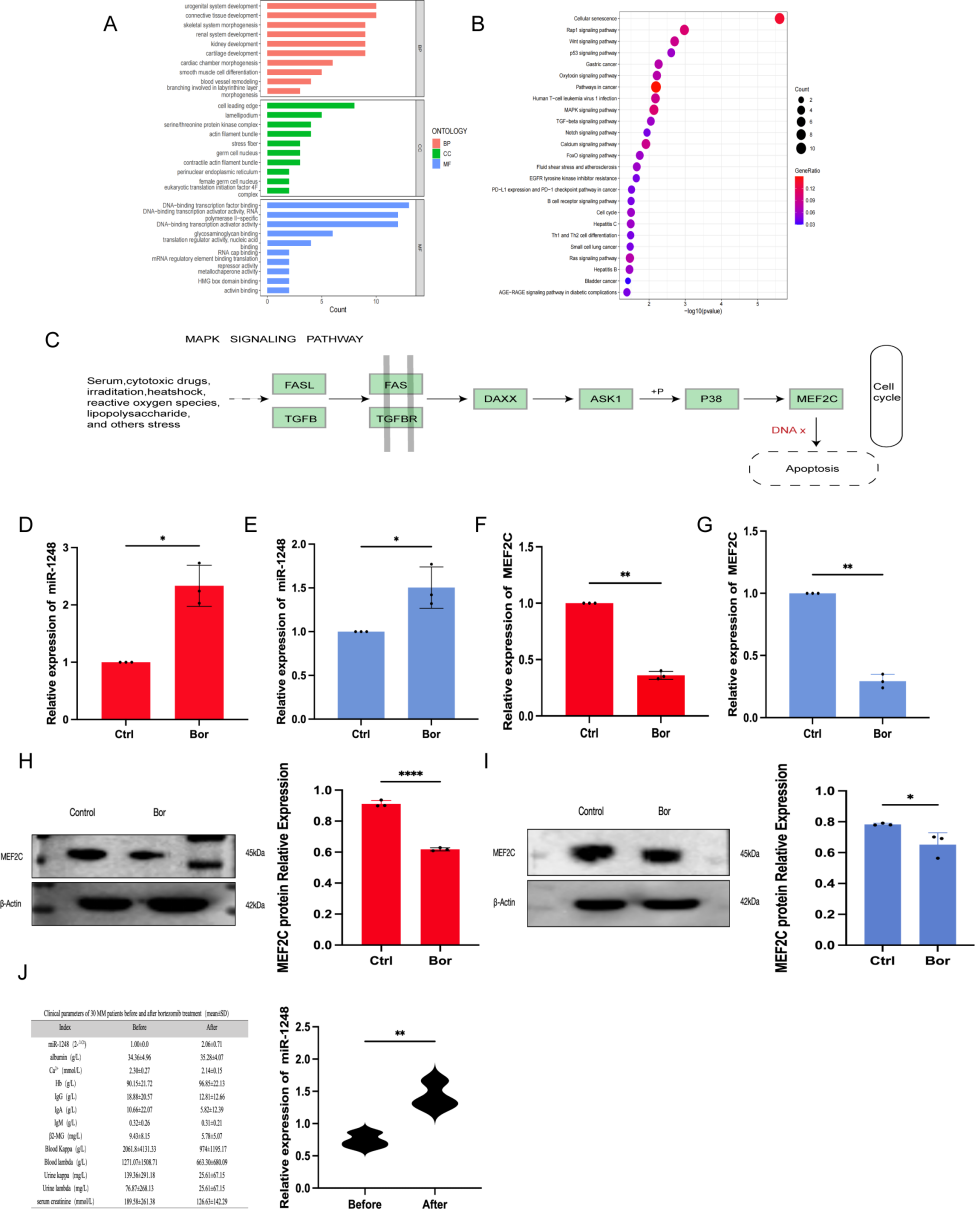
Bortezomib upregulates miR-1248 and downregulates its target MEF2C in multiple myeloma cells. **(A)** Predicted biological functions of common target genes of miR-1248. **(B, C)** KEGG pathway enrichment analysis of co-targeted mRNAs, highlighting the MAPK signaling pathway and identifying MEF2C as a key target. **(D, E)** Expression of miR-1248 in RPMI8226 **(D)** and U266 **(E)** cells after 24 h of bortezomib treatment. **(F, G)** MEF2C mRNA expression in RPMI8226 **(F)** and U266 **(G)** cells following bortezomib treatment. **(H–I)** MEF2C protein expression in RPMI8226 **(H)** and U266 **(I)** cells after bortezomib treatment. **(J)** Plasma miR-1248 levels in 30 patients with newly diagnosed multiple myeloma before and after one cycle of bortezomib-based (VCD) chemotherapy, along with clinical parameters.

Integrating TargetScan, miRDB, and our mRNA-seq data identified 123 common predicted target genes. KEGG pathway analysis showed enrichment in the MAPK signaling pathway. Given the known role of MAPK signaling in apoptosis and cellular response to bortezomib, we focused on this pathway. Among MAPK-related genes, we prioritized MEF2C because its mRNA level decreased after bortezomib treatment, consistent with an inverse relationship to miR-1248 upregulation (**Figures 1A–C**).

Validation experiments confirmed that bortezomib significantly increased miR-1248 and decreased MEF2C mRNA/protein in RPMI8226 and U266 cells (P<0.05, **Figures 1D–I**). Clinically, plasma miR-1248 levels were elevated in 30 MM patients after one cycle of bortezomib-based therapy (VCD regimen) compared to baseline (P<0.05, **Figure 1J**) (**Table 2**). Correlation with treatment response was not performed due to limited sample size and follow-up.

**Table 2:** Clinical parameters of 30 MM patients before and after bortezomib treatment(mean ± SD).

Index	Before	After
miR-1248(2-^△Ct^)	1.00±0.0	2.06±0.71
albumin(g/L)	34.36±4.96	35.28±4.07
serum creatinine(mmol/L)	189.58±261.38	126.63±142.29
Ca^2+^(mmol/L)	2.30±0.27	2.14±0.15
β2-MG(mg/L)	9.43±8.15	5.78±5.07
Hb(g/L)	90.15±21.72	96.85±22.13
IgG(g/L)	18.88±20.57	12.81±12.66
IgA(g/L)	10.66±22.07	5.82±12.39
IgM(g/L)	0.32±0.26	0.31±0.21
Blood Kappa(g/L)	2061.8±4131.33	974±1195.17
Blood lambda(g/L)	1271.07±1508.71	663.30±680.09
Urine kappa(mg/L)	139.36±291.18	25.61±67.15
Urine lambda(mg/L)	76.87±268.13	45.04±128.69

### miR-1248 directly binds to MEF2C 3′UTR and affects total p38 protein levels

**Figure 2:**
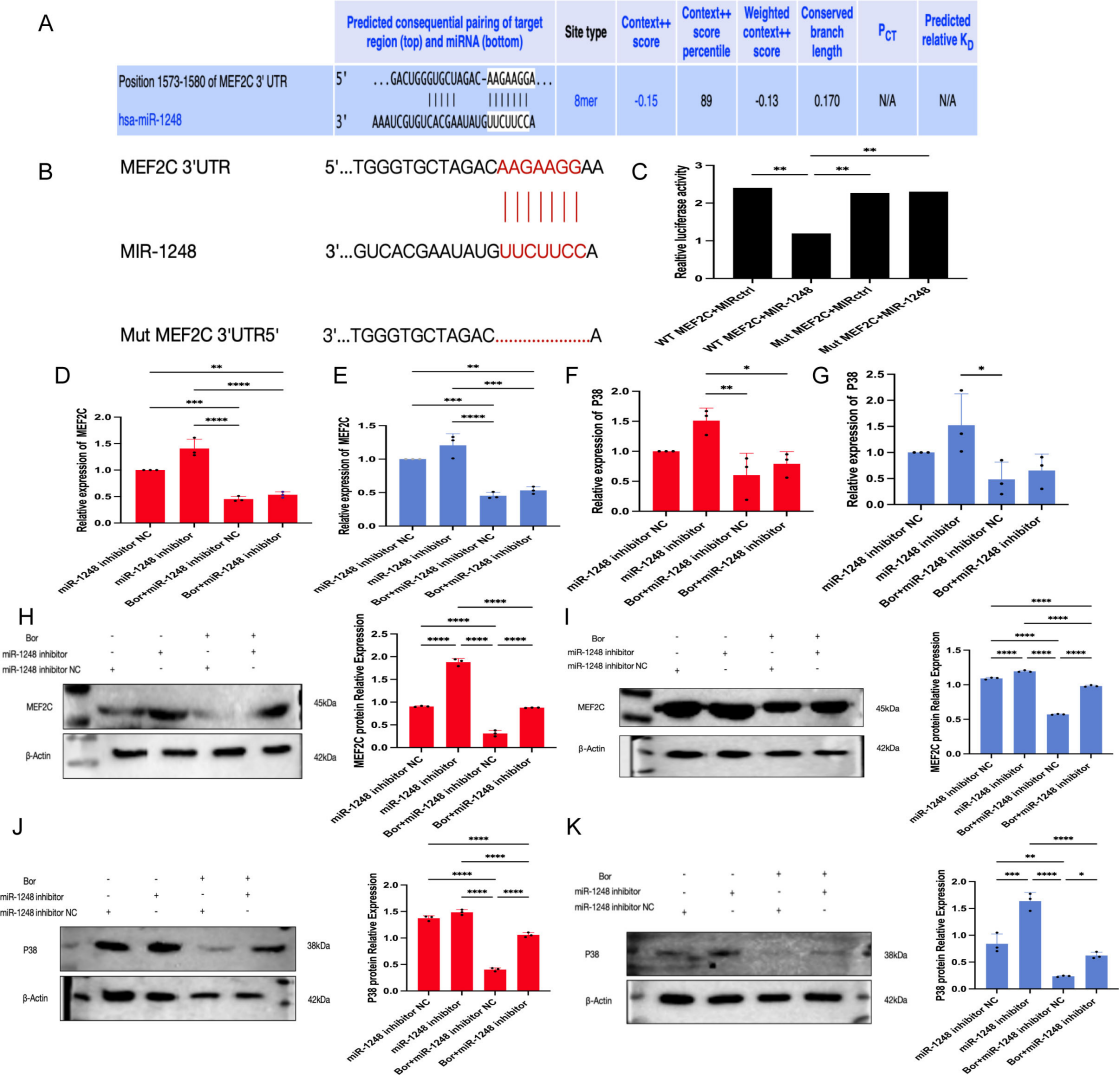
Validation of direct targeting of MEF2C by miR-1248 in multiple myeloma cells, and analysis of MEF2C and p-38 expression following miR-1248 inhibition. **(A)** Prediction of miR-1248 binding to the 3′-UTR of MEF2C. **(B, C)** Schematic illustration of miR-1248 binding to MEF2C, with mutation sites indicated in red; luciferase activity in 293T cells co-transfected with wild-type or mutant MEF2C 3′-UTR reporter plasmids together with miR-1248 mimic or control. **(D, E)** MEF2C mRNA expression in RPMI-8226 **(D)** and U266 **(E)** cells after transfection with miR-1248 inhibitor or inhibitor negative control. **(F, G)** p-38 mRNA expression in RPMI-8226 **(F)** and U266 **(G)** cells under the same conditions. **(H, I)** MEF2C protein expression in RPMI-8226 **(H)** and U266 **(I)** cells after transfection with miR-1248 inhibitor or inhibitor negative control. **(J, K)** p-38 protein expression in RPMI-8226 **(J)** and U266 **(K)** cells under the same conditions.

TargetScan predicted a putative miR-1248 binding site within the MEF2C 3′UTR (**Figure 2A**). We constructed luciferase reporters containing wild-type or mutant (four point mutations in the seed region) sequences. In 293T cells, miR-1248 mimics significantly reduced wild-type activity but did not affect the mutant (P < 0.05), supporting direct binding (**Figures 2B, C**).

Transfection with miR-1248 inhibitor reversed bortezomib-induced MEF2C suppression and restored total p38 levels (**Figures 2D–K**). p38-MAPK signaling is primarily regulated through phosphorylation; our data show changes in total p38 only and do not directly demonstrate pathway activity.

### miR-1248 modulates bortezomib-induced changes in autophagy-associated markers and lysosomal compartments

**Figure 3:**
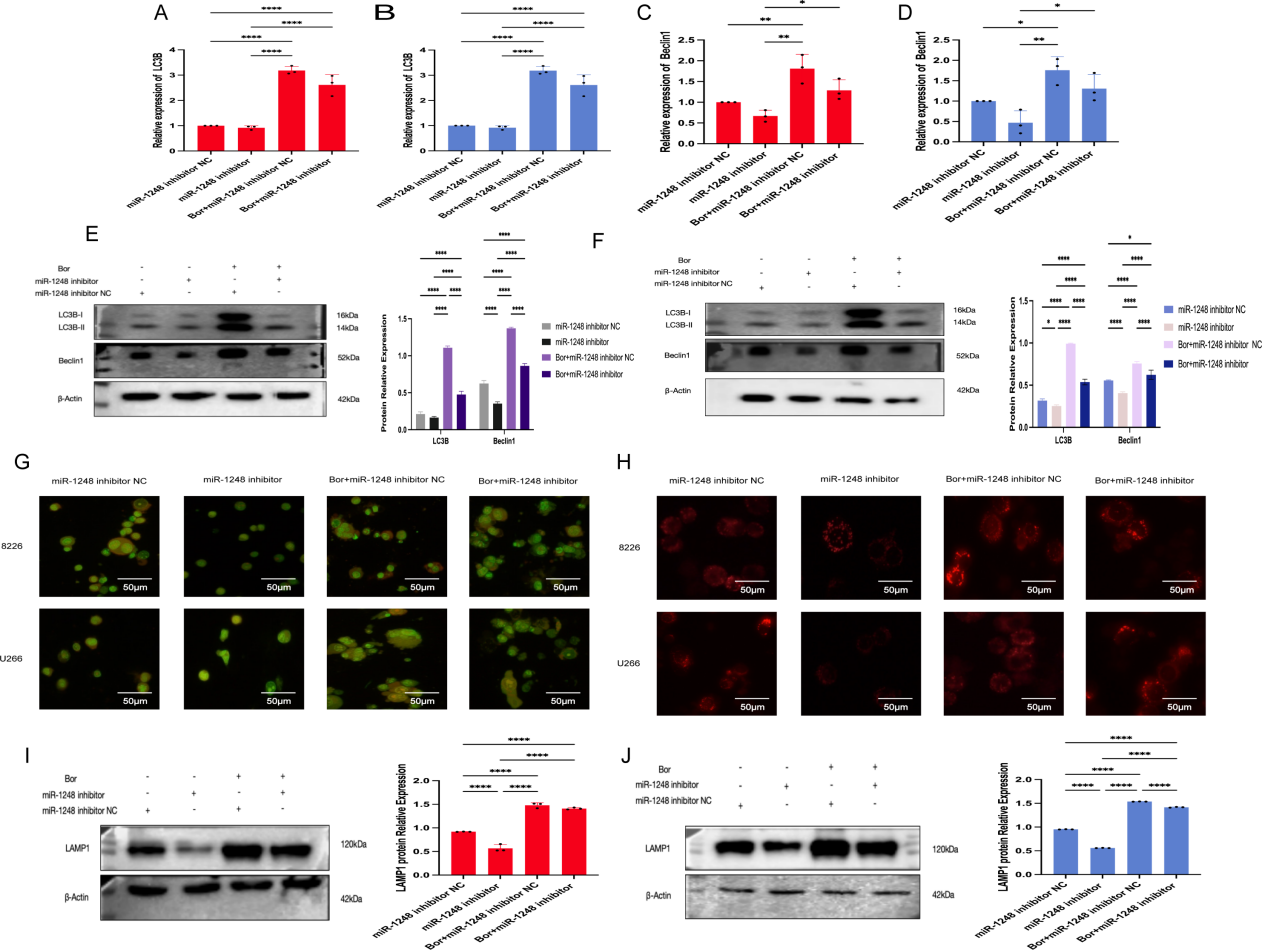
Analysis of autophagic flux in multiple myeloma cells following transfection with miR-1248 inhibitor or inhibitor negative control (NC). **(A, B)** LC3 mRNA expression in RPMI-8226 **(A)** and U266 **(B)** cells after transfection with miR-1248 inhibitor or inhibitor NC. **(C, D)** Beclin1 mRNA expression in RPMI-8226 **(C)** and U266 **(D)** cells under the same conditions. **(E, F)** LC3 and Beclin1 protein expression in RPMI-8226 **(E)** and U266 **(F)** cells following transfection with miR-1248 inhibitor or inhibitor NC. **(G)** Autophagosome expression in RPMI-8226 and U266 cells. **(H)** Lysosomal expression changes in RPMI-8226 and U266 cells. **(I, J)** LAMP1 protein expression in RPMI-8226 **(I)** and U266 **(J)** cells, with representative protein bands and relative expression levels.

miR-1248 inhibition partially attenuated bortezomib-induced upregulation of LC3B and Beclin1 in both cell lines (**Figures 3A–F**). Acridine orange staining showed that bortezomib increased acidic vesicles, an effect reduced by miR-1248 inhibition (**Figure 3G**). Lysosomal content (Lyso-Tracker) and LAMP1 expression showed similar patterns (P < 0.05, **Figures 3H–J**). Together, these results indicate that miR-1248 modulates bortezomib-induced changes in autophagy-related protein expression and lysosomal compartments.

### MEF2C overexpression attenuates bortezomib-induced apoptosis and reduces the upregulation of autophagy-associated markers

**Figure 4:**
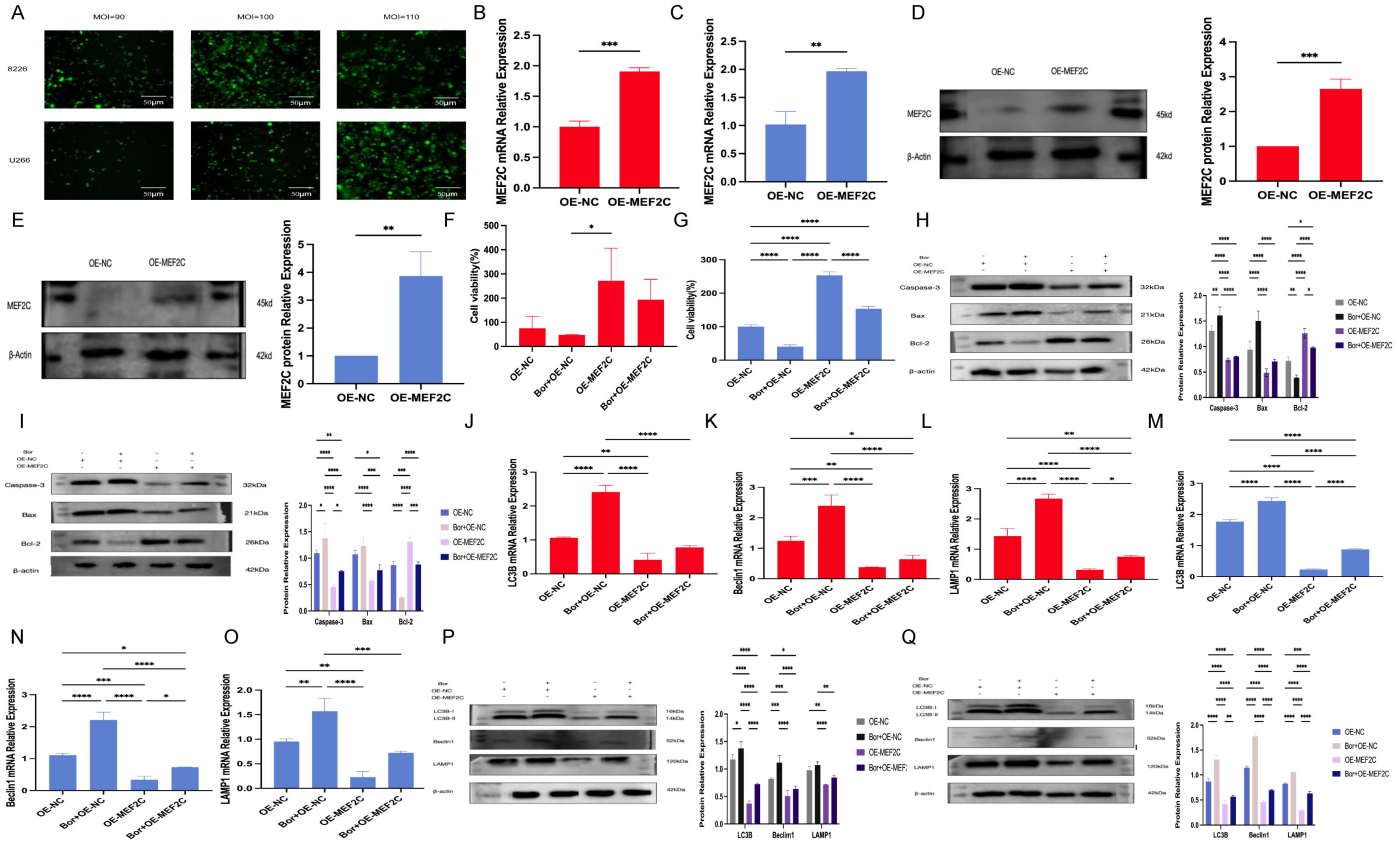
MEF2C overexpression attenuates the inhibitory effect of bortezomib on multiple myeloma cell proliferation and regulates the expression of autophagy-related factors. **(A)** Transduction efficiency of MEF2C-overexpressing lentivirus in RPMI-8226 and U266 cells. **(B, C)** MEF2C mRNA expression in RPMI-8226 **(B)** and U266 **(C)** cells following MEF2C overexpression. **(D, E)** MEF2C protein expression in RPMI-8226 **(D)** and U266 **(E)** cells under the same conditions. **(F, G)** Inhibitory effect of bortezomib on cell proliferation in RPMI-8226 **(F)** and U266 **(G)** cells transduced with MEF2C-overexpressing lentivirus. **(H, I)** Expression of apoptosis-related proteins in RPMI-8226 **(H)** and U266 **(I)** cells. **(J–O)** mRNA expression of autophagy-related factors in RPMI-8226 **(J–L)** and U266 **(M–O)** cells. **(P, Q)** Protein expression of autophagy-related factors in RPMI-8226 **(P)** and U266 **(Q)** cells.

RPMI8226 and U266 cells were transduced with MEF2C-overexpressing lentivirus (MOI = 100 and 110, respectively; **Figure 4A**). Stable clones showed significant upregulation of MEF2C mRNA and protein (P < 0.05, **Figures 4B–E**). MEF2C overexpression significantly reduced bortezomib-induced apoptosis (CCK-8 and flow cytometry) and blunted the changes in Bax, cleaved Caspase-3, and Bcl-2 (P < 0.05, **Figures 4F–I**).

Bortezomib-induced upregulation of LC3B, Beclin1, and LAMP1 was also suppressed by MEF2C overexpression (**Figures 4J–Q**). Thus, MEF2C overexpression protects against bortezomib-induced apoptosis and counteracts the bortezomib-induced increase in these autophagy-related markers.

### Inhibition of miR-1248 attenuates bortezomib-induced apoptosis and autophagy in multiple myeloma xenografts

**Figure 5:**
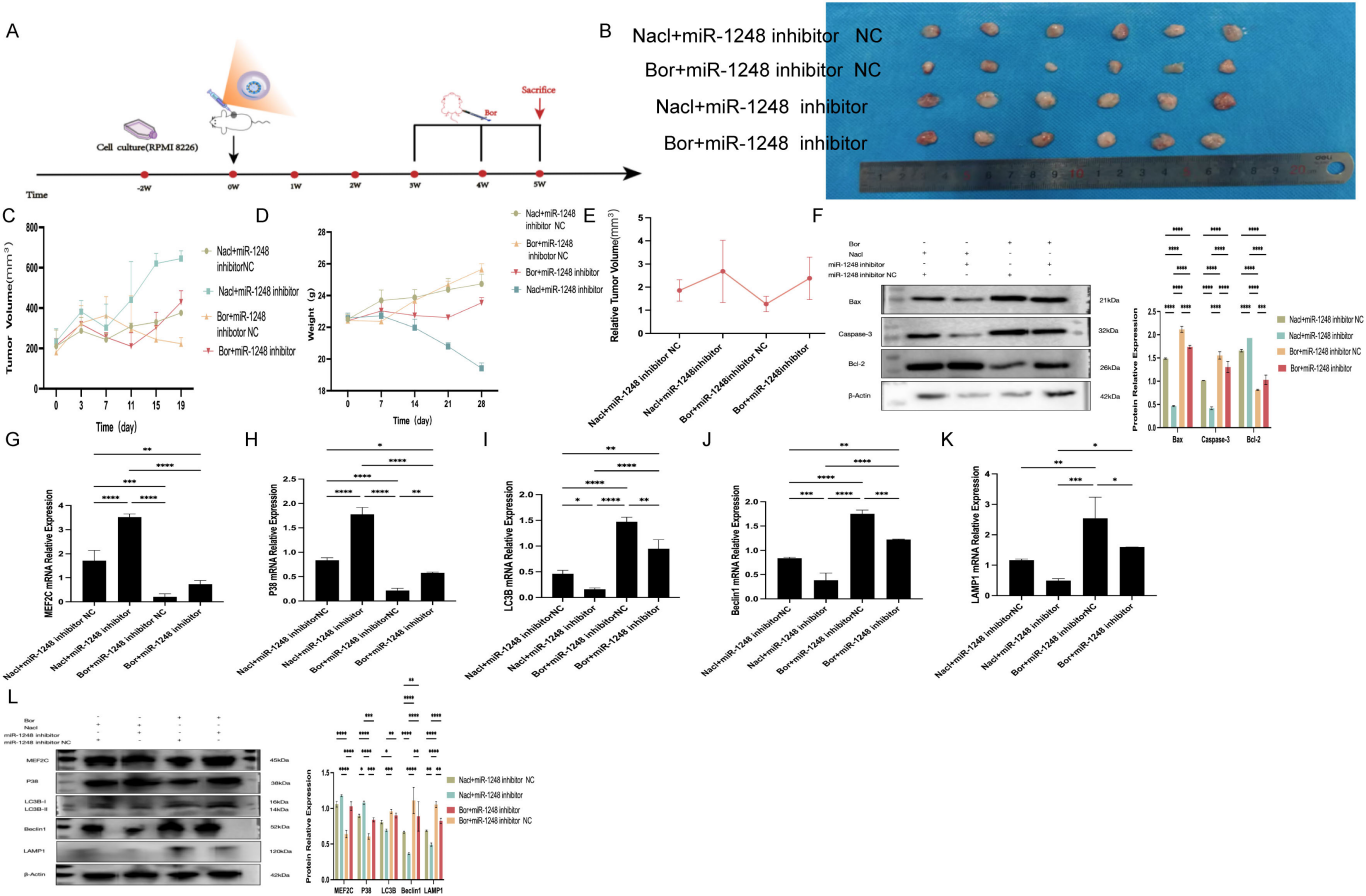
Expression levels of apoptosis- and autophagy-related factors *in vivo*. **(A, B)** *In vivo* experimental design and representative images of xenograft tumors. **(C–E)** Tumor volume, relative tumor size, and tumor inhibition rates in the four treatment groups. **(F)** Expression levels of apoptosis-related factors (Bax, Caspase-3, Bcl-2) in each group. **(G–K)** mRNA expression of MEF2C, p-38, and autophagy-related factors. **(L)** Protein expression of MEF2C, p-38, and autophagy-related factors.

RPMI8226 cells pre-transfected with miR-1248 inhibitor or NC were injected subcutaneously into nude mice (formation rate 93%). On day 14, 24 tumor-bearing mice were randomized into four groups (n=6, **Figures 5A, B**). At the end of the experiment, mean tumor volume in the Bor+NC group was 185 ± 42 mm³, while the Bor+miR-1248 inhibitor group showed significantly larger tumors (412 ± 67 mm³, P < 0.01), with inhibition rates of 58% *vs* 21% (**Figures 5C–E**).

Western blot showed that miR-1248 inhibition decreased Bax and cleaved Caspase-3 and increased Bcl-2 under both NaCl and bortezomib conditions (P < 0.05, **Figure 5F**), indicating partial abrogation of bortezomib’s pro-apoptotic effect. Bortezomib-induced upregulation of LC3B, Beclin1, and LAMP1 was attenuated by miR-1248 inhibition, while bortezomib-suppressed MEF2C and total p38 were partially restored (P < 0.05, **Figures 5G–L**). These findings suggest that miR-1248 inhibition reduces bortezomib-induced changes in autophagy-related markers, possibly via restoring MEF2C and total p38.

In conclusion, our data present a working model wherein miR-1248 binds to the 3′UTR of MEF2C and negatively regulates total p38 levels, correlating with bortezomib-induced apoptosis and changes in autophagy-related markers in multiple myeloma cells. The observation that miR-1248 inhibition or MEF2C overexpression alleviates bortezomib-induced cytotoxicity suggests that this axis contributes to cellular response to bortezomib. Modulating the miR-1248/MEF2C/p38-MAPK axis may represent a potential strategy to enhance bortezomib sensitivity in multiple myeloma. 

## Discussion

In this study, we identified miR-1248 as a bortezomib-responsive miRNA in multiple myeloma. RNA-seq and clinical sample validation showed that bortezomib treatment increased miR-1248 expression, accompanied by changes in MM cell proliferation and apoptosis. Mechanistically, miR-1248 directly targeted MEF2C. This was associated with reduced total p38 levels and altered expression of autophagy-related markers (LC3B, Beclin1, LAMP1) — a function not previously reported in MM. Unlike its pro-tumor role in lung cancer, miR-1248 contributed to bortezomib sensitivity in MM, distinguishing it from other miRNAs that modulate bortezomib response through different mechanisms. These findings were supported by *in vivo* xenograft experiments.

Autophagy plays a dual role in myeloma cells: it supports survival under basal conditions but may lead to cell death when excessively activated (20, 23–40). Previous studies have shown that various therapeutic agents can shift this balance (41–60). Our results indicate that miR-1248-mediated downregulation of MEF2C is linked to changes in autophagy-related markers upon bortezomib treatment. Inhibiting miR-1248 attenuated the bortezomib-induced increases in LC3B, Beclin1, and LAMP1, while partially restoring MEF2C and total p38 expression. However, we measured only total p38, not phosphorylated p38; therefore, we cannot conclude whether the p38-MAPK pathway is activated or inactivated. Likewise, our staining assays (acridine orange and Lyso-Tracker) detect acidic vesicular compartments and lysosomal changes, not autophagic flux. The directionality between MEF2C and p38 in MM cells also remains unresolved — our data show that both change coordinately with miR-1248 modulation, but which acts upstream requires further investigation.

Our experiments evaluated acute bortezomib response rather than acquired resistance. Thus, we interpret miR-1248 as a modulator of bortezomib sensitivity, not a mediator of resistance. We hypothesize that restoring miR-1248 expression might sensitize MM cells to bortezomib, but this needs direct testing using miR-1248 mimics in dose-response and long-term survival assays. Clinically, we observed that plasma miR-1248 levels increased after one cycle of VCD therapy (a bortezomib-containing regimen). This finding is preliminary. Whether baseline miR-1248 levels predict treatment response or outcome remains to be determined in larger, prospectively followed cohorts. Other limitations include the lack of RNA pull-down to directly demonstrate the miR-1248-MEF2C interaction, the absence of Annexin V flow cytometry for apoptosis, and the use of immortalized cell lines without validation in primary MM cells. The xenograft study used a single model without a therapeutic intervention arm, and our experiments were performed only with bortezomib monotherapy, whereas current clinical regimens often combine bortezomib with other agents. The clinical sample size is modest (n=30) and single-center with no prognostic follow-up.

**Figure 6:**
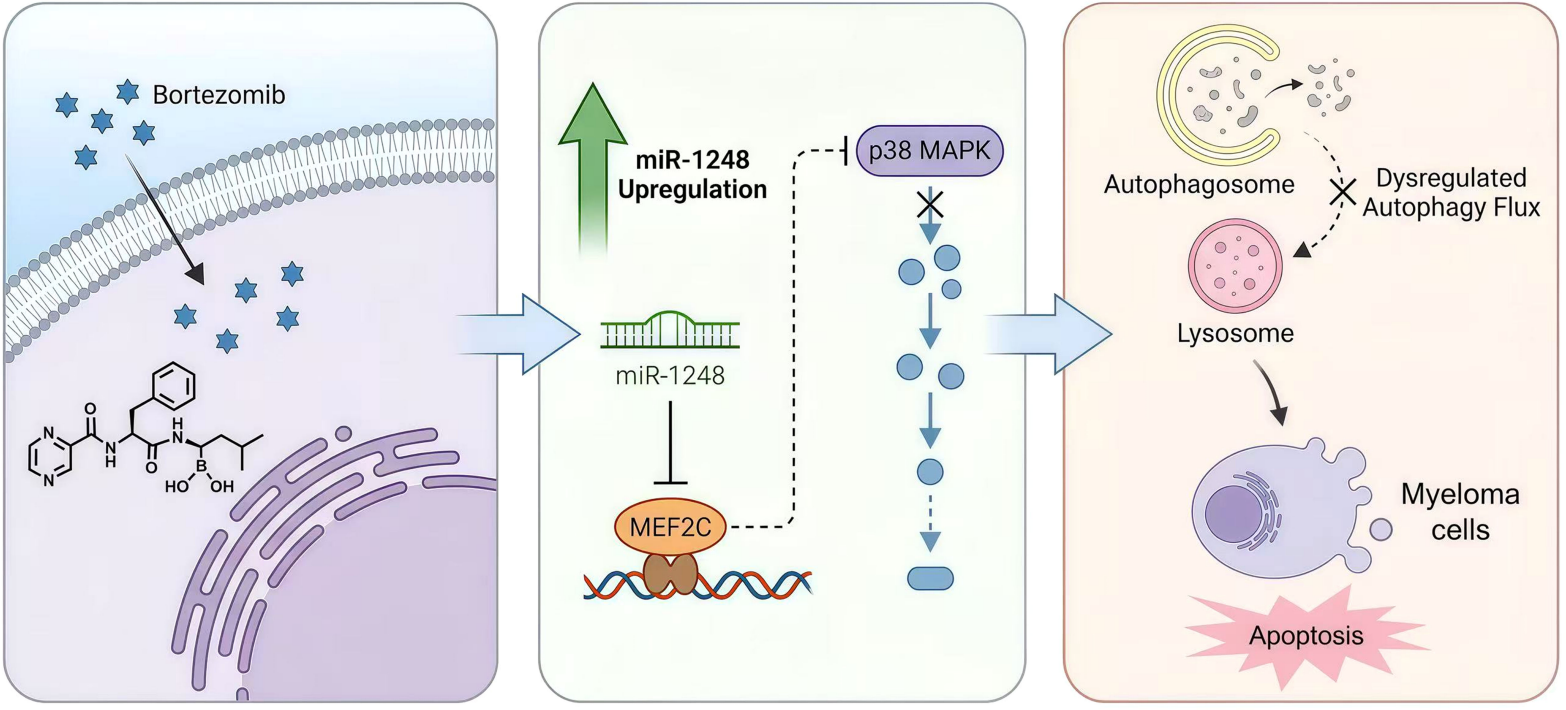
Schematic model of the proposed miR-1248/MEF2C/p38 axis in bortezomib-treated multiple myeloma cells. Bortezomib upregulates miR-1248, which directly targets the 3′-UTR of MEF2C, leading to decreased MEF2C expression, accompanied by reduced total p38 levels and altered autophagy-related markers (including LC3B, Beclin1, and LAMP1). These coordinated changes are associated with enhanced bortezomib sensitivity. Arrows indicate activation or promotion; blunted lines indicate inhibition.

Based on the above findings, we propose a schematic working model (**Figure 6**) in which bortezomib-induced miR-1248 upregulation directly targets MEF2C, accompanied by reduced total p38 expression and altered autophagy-related markers, collectively contributing to enhanced bortezomib sensitivity. Collectively, our results provide a biological rationale for further investigation of this miR-1248–MEF2C–p38 axis as a potential strategy to improve bortezomib response in MM.

## Data Availability

The original contributions presented in the study are included in the article/supplementary material. Further inquiries can be directed to the corresponding author.
